# Transcription of the apicoplast genome

**DOI:** 10.1016/j.molbiopara.2016.07.004

**Published:** 2016

**Authors:** R.E.R. Nisbet, J.L. McKenzie

**Affiliations:** Department of Biochemistry, University of Cambridge, Tennis Court Road, Cambridge, CB2 1QW, UK

**Keywords:** Apicoplast, Chloroplast, Plastid, Apicomplexa, Transcription, Post-transcriptional processing

## Abstract

Many members of the Apicomplexa contain a remnant chloroplast, known as an apicoplast. The apicoplast encodes numerous genes, and loss of the organelle is lethal. Here, we present a summary of what is known about apicoplast transcription. Unlike plant chloroplasts, there is a single RNA polymerase, and initial transcription is polycistronic. RNA is then cleaved into tRNA, mRNA and rRNA molecules. Significant levels of antisense transcription have been reported, together with a single case of RNA editing. Polycistronic transcription is also observed in the related algae *Chromera* and *Vitrella*, which retain a photosynthetic chloroplast. Surprisingly, a polyU tail is added to *Chromera* and *Vitrella* transcripts which encode proteins involved in photosynthesis. No such tail is added to *Plasmodium* transcripts. Transcription in the Apicomplexa is remarkably similar to that seen in the chloroplast of the related peridinin dinoflagellate algae, reflecting the common evolutionary origins of the organelle.

## Highlights

• Apicoplast transcription is polycistronic.
• Antisense transcription is common.
• Significant amount of post-transcriptional processing.
• Transcription closely resembles that of the dinoflagellate chloroplast.

## Graphical abstract

**Figure:**
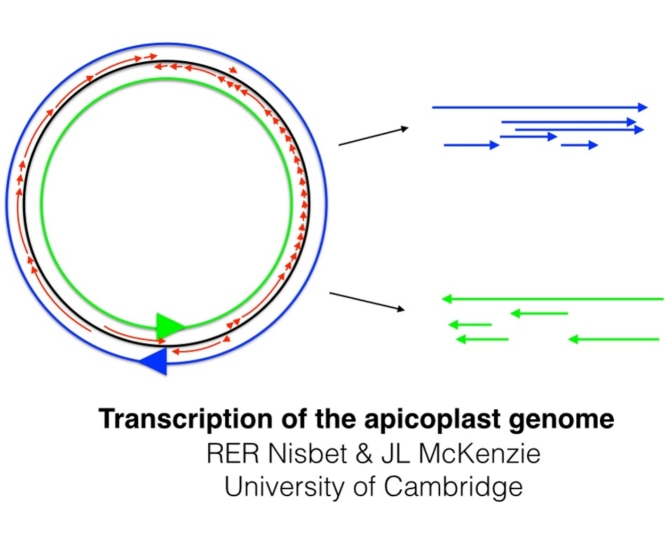

## 1 Introduction

The discovery of a remnant chloroplast in *Plasmodium* twenty years ago [1], [2], [3], [4] was a great surprise. The remnant chloroplast, known as an apicoplast, has lost the ability to carry out photosynthesis, yet remains as a small, membrane-bound organelle within every *Plasmodium* cell. The apicoplast is an essential organelle, and inhibition is lethal. The apicoplast genome resembles those found in chloroplasts, albeit much reduced at 35 kb due to the absence of genes encoding proteins involved in photosynthesis. The remaining genes encode various proteins, ribosomal RNA (rRNA) and transfer RNAs (tRNAs), which are also found in chloroplast genomes of photosynthetic species.

Chloroplasts arose from a primary endosymbiosis event between an early eukaryote and a photosynthetic bacterium, known as a cyanobacterium. The early photosynthetic eukaryotes diversified, giving rise to organisms with a range of chloroplast types. Sometime later, a secondary endosymbiosis event occurred where an ancestor to *Plasmodium* engulfed a photosynthetic alga. Whether this alga contained a chloroplast of red or a green origin is a matter of considerable debate, but most phylogenetic analyses favour the red alga hypothesis (for a review, see [5]). In either case, the result is the same: a chloroplast is now present in a previously non-photosynthetic eukaryotic lineage.

As a result of the evolutionary origin of the chloroplast, transcription and translation machineries within the organelle are of bacterial type. This explains why antibiotics which target transcription and translation function as antimalarials. For example, doxycycline, a commonly used malarial prophylaxis agent targets the bacterial-style 70S ribosome, while rifampicin targets the bacterial-style RNA polymerase [6]. Despite the importance of transcription and translation in the apicoplast, remarkably little is known about how these processes occur, or how they are regulated.

**Fig. 1:**
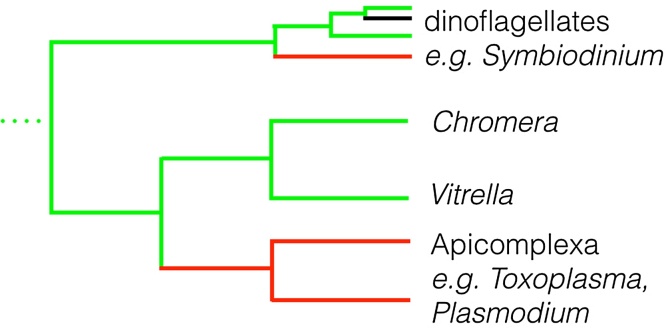
Relationship between Apicomplexa, chromerids and dinoflagellates. Representative photosynthetic species are shown in green, parasitic species are shown in red, non-photosynthetic, non-pathogenic species are shown in black. Note that there are numerous species not shown, and that the figure is not to scale.

*Plasmodium* is a member of the Apicomplexa, a group of primarily intracellular parasites, which also include *Toxoplasma* and *Eimeria*. The majority of species contain a remnant chloroplast, while those that do not (such as *Cryptosporidium*) have secondarily lost the organelle [7]. However, in the past ten years, two photosynthetic species, closely retated to Apicomplexa, have been identified: *Chromera velia* and *Vitrella brassicaformis* [8], [9]. These two species, known as the chromerids, contain a fully functional chloroplast, acquired by the same secondary endosymbiosis event as gave rise to the chloroplast parasitic apicomplexan species. Less closely related, but still harboring the same chloroplast are the dinoflagellates, photosynthetic algae often found as symbionts of coral (Fig. 1). By understanding how transcription occurs across these diverse eukaryotes, we can begin to better understand how transcription of apicoplast genes occurs in *Plasmodium* and other parasitic Apicomplexa.

### 1.1 Single RNA polymerase

Plant chloroplasts typically utilise two different RNA polymerases; a nuclear encoded polymerase (NEP) related to the single-polypeptide phage-type polymerase which generally transcribes non-photosynthesis genes and a multi-subunit plastid encoded (PEP) bacterial-type polymerase principally involved in the expression of photosynthesis genes [10], [11]. Physiological changes in chloroplast gene expression are controlled by modulating the activity of each polymerase [12], [13].

There is no evidence for the presence of a phage-type chloroplast polymerase outside the land plant lineage. Although phage-type polymerases are present in the nuclear genomes of the dinoflagellate algae and apicomplexa [14], [15] they are predicted to be targeted to the mitochondria to ensure transcription of mitochondrial genes [16]. Thus, the bacterial-type RNA polymerase is solely responsible for transcription of the apicoplast genome, and the chloroplast genomes of *Chromera, Vitrella* and dinoflagellates.

The plastid RNA polymerase consists of 5 subunits; two α (*rpoA)*, β (*rpoB), β*’ (*rpoC)* and ω *(rpoD)*. The *rpoB* and *rpoC* RNA polymerase subunits are encoded on the apicoplast genome. The *rpoC* gene of apicoplast genomes lacks the intron found in many species and is instead split into *rpoC1* and *rpoC2* [4]. In addition, the *Plasmodium rpoC2* contains a frameshift mutation (one extra nucleotide) that results in the presence of a stop codon. This is presumably resolved at translation level. In-frame stop codons are also found in *Toxoplasma rpoC1* and *Eimeria rpoC2*, which are likely to encode tryptophan [17], [18]. (Note that in the original annotation, *Plasmodium falciparum* apicoplast is mis-labelled as *rpoD*, and this has been carried forward to other accessions in other strains as well (e.g. GenBank X95275, PlasmoDB PFC10_API0017)). The *rpoA* gene is encoded on the nuclear genome.

Due to the massive gene transfer from dinoflagellate chloroplasts to the nucleus, all genes encoding the dinoflagellate chloroplast RNA polymerase are located in the nuclear genome [19].

Plastid encoded polymerase (PEP) promoters in plant chloroplasts are characterised by consensus sequences that resemble bacterial promoter sequences [20]. The nuclear genomes of land plants generally contain multiple chloroplast-targeted sigma factors, and there is evidence each is differentially regulated and target different chloroplast promoters [20], [21]. So far, no apicoplast targeted sigma factors (*rpoD)* have been identified, and it seems likely that there will be only a few sigma factor proteins, rather than many.

No clear PEP promoter sequences have been identified in *Plasmodium* [22]. It is possible that apicoplast promoter sequences are so divergent from bacterial promoters that the algorithms for the prediction of bacterial promoters are not able to identify promoter sequences within apicoplast genomes. Alternatively, it could be that transcription initiates in the small unsequenced region around the *tRNA-Ile* gene, between the two inverted repeats. This would account for both the long, polycistronic transcripts seen (see below) as well as the absence of canonical promoter sequences [1], [22], [23], [24]. Although recognisable promoter sequences have been identified in *Chromera*, these do not occur upstream of each gene, and have not been confirmed experimentally [25].

### 1.2 Transcription in the non-photosynthetic apicoplast

*Plasmodium* apicoplast genes have long been known to be transcribed. Transcription of the ribosomal RNA (rRNA) genes has been shown to be polycistronic, and primary transcripts most likely encode both SSU rRNA and the adjacent tRNA [24], [23]. Similar, long polycistronic transcripts have been identified through northern blotting of several loci, many of which have subsequently been confirmed through RT-PCR [23], [24], [26], [27], [28]. Indeed, for every apicoplast gene tested to date, a polycistronic transcript has been identified.

Northern blotting carried out on total *Plasmodium* RNA revealed the presence of multiple large transcripts (of 15, 12.5, 11.5 and 7.8 kb), encoding both *rpoB* and *rpoC* [27]. These blots also showed significant smeary hybridization, presumably representing smaller transcripts of varying lengths [27]. The longest transcript, of 15 kb represents about 45% of the apicoplast genome. Northern analyses of SSU rRNA also indicate the presence of multiple transcripts, some of which are larger than the mature SSU rRNA [24]. In addition, RNAse protection experiments suggested that SSU rRNA is co-transcribed with the adjacent tRNA [24]. Together, these results suggest that the primary form of transcription in the apicoplast is polycistronic.

If the primary transcript produced in the apicoplast is polycistronic, it must follow that the RNA is cleaved to release tRNAs, rRNAs and mRNAs. Indeed, tRNA rRNA and mRNA molecules corresponding to individual genes have been identified through northern blotting [23], [26], [27]. The tRNA-Leu gene includes an intron, which is spliced out [26]. The intron is likely to be self-splicing, as occurs with the tRNA-Leu intron in red algae and green plants as well as the ancestral cyanobacterium [29].

The cleavage sites for transcripts encoding several apicoplast genes has been identified using circular RT-PCR, a process which identifies only processed RNA [23]. This has revealed that there are numerous, specific cleavage sites around the genome. These are often, but not always, associated with the exact 3′ or 5′ end of tRNA molecules. This is not dis-similar to the so-called ‘punctuation processing’ seen in mitochondria, where transcripts are cleaved at sites adjacent to tRNA molecules [30]. Analysis of *Plasmodium* transcripts show that that many cleavage site are associated with an adjacent UUAUA motif. This suggests that the mechanism for cleavage may be similar for all transcripts, and may be associated with a specific protein [23].

**Fig. 2:**
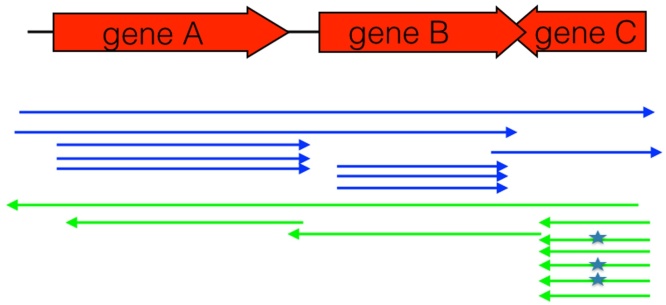
Transcription of apicoplast genes. Long, primary polycistronic transcripts are synthesized which are then cleaved into smaller RNA fragments. Both sense and antisense transcripts are produced. Protein-coding genes are shown as red arrows, and tRNA genes as letters. Blue arrows indicate sense RNA transcripts, green arrows indicate antisense RNA transcripts. Note that this does not represent an actual locus, but is schematic to show examples of different types of RNA processing.

Fig. 2 shows the current model for apicoplast transcription: a long, primary transcript which is cleaved to form tRNA, mRNA and rRNA molecules. Alternative cleavage sites are present, allowing the production of mRNA from overlapping genes.

It is not known if extensive polycistronic transcription occurs in other parasitic Apicomplexa, or if apicoplast genes are transcribed individually. A large-scale microarray experiment carried out by Bahl et al. [31] revealed that the presence of transcripts covering the entire *Toxoplasma* apicoplast genome. Transcript levels varied across the apicoplast genome, with some genes being highly expressed and others expressed at a much lower level. However, due to the nature of microarray analysis, it is not possible to determine if this is due to multiple promoter regions, and thus monocistronic transcription, or polycistrionic transcription followed by cleavage producting individual mRNA/tRNA/rRNA molecules.

### 1.3 Transcription in Chromerid algae

To date, only two species of Chromerid algae have been identified, *Chromera velia* and *Vitrella brassicaformis.* These are the closest photosynthetic relatives of the Apicomplexa [8], [9]. Chloroplast genomes from both species have been sequenced and both contain genes necessary for photosynthesis [32]. The *Chromera* chloroplast genome is a single linear chromosome, whereas the *Vitrella* chloroplast genome is a single circular chromosome, similar to that found in the parasitic apicomplexans [32], [33]. Gene order has been altered, although genes encoding functionally related proteins remain together.

Polycistronic transcription is widespread in both *Chromera* and *Vitrella* chloroplasts [25], [33]. *Chromera* RNA-seq data reveal that transcription levels are highest for *psbA,* which encodes a core protein in photosynthesis [33].

*Chromera* genes *psaA* and *atpB* are broken into two fragments, as occurs in some other photosynthetic chloroplast lineages. The two fragments are independently transcribed, translated and then assembled into functional Photosystem I and ATP synthase proteins [33]. In contrast, in other chloroplast lineages with split genes, the RNA transcripts are spliced to form a single transcript which is then translated into the functional protein [34].

### 1.4 Transcription in dinoflagellate algae

Dinoflagellates, the sister group to the Apicomplexa, are a large and diverse group of algae. The ‘typical’ dinoflagellate (peridinin dinoflagellates, named after the major accessory pigment in photosynthesis) contains a fully functional, photosynthetic chloroplast. The chloroplast genome is fragmented into multiple plasmid-like minicircles, each encoding 0–3 genes [35], [36]. Each minicircle is approximately 3 kb in length, and contains a well-conserved, species-specific core region. Northern analyses have shown that all minicircles are transcribed, giving rise to both gene-specific mRNA molecules and long, polycistronic RNA molecules. The long, polycistronic molecules are approximately the length of the whole minicircle, suggesting that the whole chloroplast genome is transcribed. These polycistronic molecules are then cleaved into gene-sized mRNA fragments [37], [38], [39], [40].

The origin of transcription for each minicircle is likely to be within the core region, which would explain why the coding region is always in the same orientation with respect to the core. However, no identifiable −35 and −10 sequences upstream of transcriptional start sites have been identified [41].

Many dinoflagellate species have lost their chloroplasts, and are no longer photosynthetic. Other species have replaced the original chloroplast with a so-called tertiary plastid, as a result of an endosymbiosis with another alga, such as a diatom or haptophyte [42], [43]. The chloroplast genomes in these dinoflagellate species are more conventional, resembling the donor chloroplast and are thus not fragmented.

### 1.5 Antisense transcription

Surprisingly, an analysis of *Toxoplasma* apicoplast transcription revealed that both DNA strands are transcribed, giving rise to significant levels of antisense transcripts. High levels of antisense transcription often, but not always corresponded with high levels of sense transcription [31]. These results have been confirmed by the discovery of antisense transcripts in *Plasmodium* [23], including the presence of long, polycistronic antisense transcripts. These transcripts cover protein-encoding, tRNA and rRNA genes, and may be processed at the same sites as the corresponding sense transcripts, as shown in Fig. 2.

It is not clear why antisense transcripts are present. In plant chloroplasts, antisense transcripts have been shown to provide a role in the regulation of gene expression [44], [45]. Alternatively, their presence may simply be a result of read- through transcription [46]. Genes on the apicoplast genome are arranged in two major operons, each in opposing orientations, so it is possible that read-through transcription from one operon results in the production of antisense transcripts for the other operon. This would be the simplest explanation, but does not explain the presence of conserved processing sites [23].

In a conventional (peridinin) dinoflagellate chloroplast, all genes on a single minicicle are encoded in the same orientation. It is therefore not possible to produce antisense transcripts from read-through transcription, as could occur in the Apicomplexa chloroplast. Thus, if antisense transcripts are present in peridinin dinoflagellates, they must be specifically transcribed. Recently, antisense chloroplast transcription has been discovered in the dinoflagellate *Karenia mikimotoi* [47]. It should be noted that this is a tertiary chloroplast (i.e. a replacement chloroplast), and thus is not ancestral. It will be interesting to determine if antisense transcription occurs in peridiniun dinoflagellates.

Given the common evolutionary origins of dinoflagellate and apicomplexa chloroplasts it therefore seems likely that if antisense transcription is not accidental, the function of antisense transcripts would be the same in both lineages.

### 1.6 Post-transcriptional processing

#### 1.6.1 Addition of a polyU tail

In a feature that is unique to the chromerid algae and dinoflagellates, a post-transcriptional polyU tail is added to many chloroplast transcripts (Table 1). The first reports came from peridinin dinoflagellates, where chloroplast transcripts (all encoding photosynthesis genes) were found to be post-transcriptionally modified by the addition of a polyU tail [39], [48]. The addition of a polyU tail has also been reported in *Chromera* and *Vitrella*, the two most closely related photosynthetic organisms to Apicomplexa [25], [32], [33]. Here, the polyU tail is preferentially added to transcripts encoding proteins involved in photosynthesis, while is the majority of non-photosynthesis transcripts do not have a polyU tail. (Transcripts that receive polyU tail addition in *Chromera*: 22/25 photosynthesis genes, 16/38 non-photosynthesis genes [25]). There is no evidence for polyU tail addition in *Plasmodium* (Table 1) [25]. It is not possible to determine if polyU tail addition was once applied to all chloroplast transcripts and subsequently lost in non-photosynthesis genes, or if the specific polyU tail addition has always been specific to photosynthesis genes. However, the linking of a loss of polyU and the loss of photosynthetis is appealing. It is is also unclear why polyU tails are added. It may be to increase transcript stability, as is the case for the addition of polyA tails to nuclear mRNA transcripts.

#### 1.6.2 RNA editing

RNA editing of plant chloroplast transcripts is common, and may play a role in the regulation of gene expression, or as a method of increasing sequence diversity [49], [50]. RNA editing also occurs in dinoflagellate chloroplast transcripts, though rates of editing vary from extremely high to little or none [19], [48], [51], [52], [53]. Editing has not been identified in *Chromera* and *Vitrella* [33]. However, a single case of RNA editing has recently been reported in the *Plasmodium rpl2* gene (Table 1) [23]. It is not clear if the ancestral chloroplast had RNA editing, and it has been lost in various lineages, or if the apparently random distribution of RNA editing is due to multiple gain of function events.

## 2 Summary

At first glance, transcription in the apicoplast looks to be unusual, with no promoters, polycistronic transcripts, antisense transcripts, RNA editing and conserved processing sites. However, when compared to chloroplast transcription in *Chromera, Vitrella* and dinoflagellate algae, many of these features are in fact ancestral, and are shared across many species (Table 1). The presence of so many post-transcriptional modifications may well prove to be drug targets in the ongoing fight against malaria and other diseases caused by parasitic Apicomplexa.

## Figures and Tables

**Table 1:** Summary of transcription and post-transcriptional processing events.

	polycistronic transcripts	antisense transcripts	polyU photosynthesis genes	polyU non photosynthesis genes	RNA editing
dinoflagellates	YES	?	YES	16 S and 23S	some species
*Chromera*	YES	?	YES	few	NO
*Vitrella*	YES	?	YES	few	NO
*Toxoplasma*	probably	YES	N/A	? probably no	?
*Plasmodium*	YES	YES	N/A	NO	YES,*rpl2*
